# Gastrointestinal bleeding in patients with COVID-19: an integrative review

**DOI:** 10.1590/0100-6991e-20243600-en

**Published:** 2024-04-17

**Authors:** ANA CLARA FREITAS GALVÃO SOARES DA COSTA, OLIVAL CIRILO LUCENA DA FONSECA

**Affiliations:** 1 - Faculdade de Medicina Uninassau - Recife - PE - Brasil; 2 - Hospital Universitário Oswaldo Cruz - Recife - PE - Brasil

**Keywords:** COVID-19, Gastrointestinal Hemorrhage, Endoscopy, Gastrointestinal, Therapeutics, COVID-19, Hemorragia Gastrointestinal, Endoscopia Gastrointestinal, Terapêutica

## Abstract

**Introduction:**

In 2020, the world suffered a major impact from the COVID-19 pandemic, especially due to the high transmissibility of the virus. It is a disease that predominates with respiratory manifestations, but there is involvement of the gastrointestinal tract, causing symptoms ranging from mild to more severe. Highlighting gastrointestinal bleeding, it is a symptom resulting from the involvement of the SARS-CoV-2 virus described by several reports and case series.

**Methods:**

through an integrative literature review, of a qualitative nature, works that corresponded to the eligibility criteria were selected, totaling 16 articles included in this review.

**Results:**

of the patients who manifested gastrointestinal symptoms associated with the disease, common comorbidities and clinical manifestations were identified, in addition to therapies used to treat the infection, which were predisposing factors for the development of gastrointestinal bleeding.

**Conclusion:**

The presence of gastrointestinal bleeding in patients with COVID-19 is established in the literature, since the pathophysiological mechanisms of the disease directly affect the GIT. Early recognition of symptoms and suspicion of gastrointestinal involvement allows better management of patients and complications.

## RESUMO

**Introdução:**

em 2020 o mundo sofreu um grande impacto com a pandemia pela doença COVID-19, em especial pela alta transmissibilidade do vírus. É uma doença que predomina com manifestações respiratórias, porém existe um acometimento do trato gastrointestinal, causando desde sintomas leves a mais graves. Destacando o sangramento gastrointestinal, é um sintoma decorrente do acometimento do vírus SARS-CoV-2 descrito por diversos relatos e séries de casos.

**Métodos:**

por meio de uma revisão integrativa da literatura, de caráter qualitativo, foram selecionados trabalhos que correspondessem aos critérios de elegibilidade, totalizando 16 artigos incluídos nesta revisão.

**Resultados:**

dos pacientes que manifestaram sintomas gastrointestinais associados a doença, foram identificadas comorbidades e manifestações clínicas em comum, além de terapias utilizadas como tratamento da infecção, que foram fatores predisponentes para o desenvolvimento de sangramentos gastrointestinais.

**Conclusão:**

a presença de sangramento gastrointestinal em pacientes com COVID-19 é um dado firmado na literatura, uma vez que os mecanismos fisiopatológicos da doença afetam diretamente o TGI. O reconhecimento precoce dos sintomas e a suspeita do acometimento gastrointestinal permite um melhor manejo dos pacientes e das complicações.

## INTRODUCTION

In 2019, numerous cases of pneumonia emerged in China, attributed to a new strain of the coronavirus, SARS-CoV-2, responsible for causing the disease COVID-19. In 2020, the World Health Organization declared a pandemic due to the spread of the virus around the world. The virus has a high dissemination capacity, and is predominantly respiratory^1^. However, the involvement of other organic systems, such as the nervous and gastrointestinal (GI) ones, has been reported in the literature^2^.

Regarding the manifestations of COVID-19 in the gastrointestinal tract (GIT), some pathophysiological mechanisms have already been described, especially those related to the role of angiotensin-converting enzyme 2 (ACE-2) receptor as an entry for virus penetration into gastrointestinal cells, where such molecules are highly expressed. Furthermore, the hypercoagulable state predisposed by COVID-19 can generate foci of ischemia in the GI mucosa, which generates exudation and a compensatory mechanism to reestablish perfusion, releasing inflammatory cytokines and altering the local microbiota^2^ ^-^ ^4^.

Some of the most common associated symptoms are nausea, vomiting, bleeding, anorexia, abdominal pain, and diarrhea. Hemorrhagic colitis, bloody diarrhea, and ulcerative and ischemic changes are described as rare disorders, though with greater prevalence in critically ill patients^2^ ^,^ ^3^. In the individuals who develop a more aggressive form of the disease, a combination of factors, especially GI bleeding, can trigger death^4^ ^,^ ^5^.

Bleeding of GI origin can manifest in the form of lower gastrointestinal bleeding (LGIB) or upper gastrointestinal bleeding (UGIB). This symptom may or may not be accompanied by respiratory manifestations of COVID-19, as there is description in the literature of gastrointestinal bleeding as an initial symptom of COVID-19, but also as a progression of the disease, especially in patients who develop more aggressive forms and are admitted to intensive care units (ICU)^2^ ^-^ ^4^.

It is postulated that the involvement of GI cells by COVID-19 causes inflammation through the release of cytokines, predisposes bacterial translocation through exudation, and triggers damage to the mucosa through foci of ischemia resulting from the hypercoagulable state, causing GI bleeding through primary and secondary mechanisms. Therefore, this study aims to identify the pathophysiological mechanisms that trigger gastrointestinal bleeding in patients infected with SARS-CoV-2, as well as the most used diagnostic and therapeutic mechanisms.

## METHODOLOGY

The present study is a bibliographical review of the literature, with a qualitative nature. We carried out the search in the PubMed database, using the terms “Gastrointestinal Tract AND bleeding AND COVID-19”, covering articles from 2019 to March 2023. We found 64 articles, of which we pre-selected 26, based on reading the titles and abstracts. After fully reading the pre-selected articles, we considered 16 for inclusion in this review. 

### Eligibility Criteria

**Figure 1:**
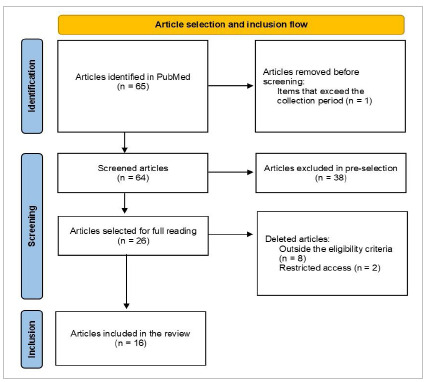
PRISMA flow diagram.

We used articles in English, with the presence of at least one of the used terms in the abstract and/or title. In the pre-selection, works that had one of the chosen term for the research in the title and/or abstract were considered. For full reading and inclusion in the review, the articles considered were prospective and retrospective studies, case reports, case series, and letters to the editor with case reports. We excluded articles that did not bring uniformity in the results found and in the statistical analysis performed, literature reviews, articles whose methodology was not detailed, and those in which gastrointestinal bleeding was neither directly nor indirectly related to SARS-CoV-2 infection. We also excluded from the analysis articles from multicenter studies that did not incorporate the results obtained between centers. The selection flow is described in the PRISMA flow diagram (Figure 1).

## RESULTS

After a careful selection, we included 16 articles in the qualitative analysis, of which two retrospective cohorts, three case series, and 11 case reports. Primary information about the cases and original studies is described in Tables 1, 2 and 3.

**Table 1:** Clinical aspects and evolution of case reports and series.

Author and year	Sex	Age	Comorbidities	GI symptoms	Diagnostic examination	Cause of bleeding	Outcome
Mohamed et al. 2021^6^	M	75	DM*	Hematochezia, hemodynamic instability	Pelvic arteriography	Bleeding in the rectal branches	DISCHARGE
Argan et al. 2021^7^	M	70	DM type 2	Rectal bleeding	CT	Perforated sealed cecal mass	DISCHARGE
	M	37	Morbid obesity	Rectal bleeding	CT	Perforated sealed cecal mass	DISCHARGE
	M	74	DM type 1	Rectal bleeding	CT	Perforated sealed cecal mass	DISCHARGE
Mitrovic et al. 2022^5^	M	33	SAH, CKD	Epigastric pain, nausea, vomiting	CT and UDE	Necrotizing pancreatitis with ruptured pseudoaneurysm	DEATH
Li et al. 2021^8^	M	91	NR	Melena and hemodynamic instability	SBCE	Jejunal and duodenal ulcers	NR
	F	83	NR	Recurrent hematochezia	SBCE	Jejunal and duodenal ulcers	NR
Yamakawa et al. 2022^9^	M	64	SAH, DM*	Diarrhea and hematochezia	UDE and LDE	Multiple ulcers in the sigmoid colon and rectum	DEATH
Kawabata et al. 2021^10^	M	70	DM, Hyperlipidemia	Hematemesis	UDE	Multiple gastric ulcers	NR
Krejčová et al. 2022^11^	NR	67	Metabolic syndrome, DM, hyperlipidemia	Massive enterorrhagia, hemodynamic instability	LDE and laparo	Ischemic ulcerative colitis	NR
Merdad et al. 2021^12^	M	23	None	Nausea and melena	CT	Perforated duodenal ulcer	DEATH
Carl et al. 2021^13^	M	Middle- age**	NR	Diarrhea, hematochezia, and nausea	LDE	Invasive enterocolitis	DISCHARGE
Jabłońska et al. 2022^14^	M	80	NR	Abdominal pain, melena, and hematochezia	Emergency laparotomy	GIST	DEATH
Carvalho et al. 2020^15^	M	71	Hypertension, depression, chronic back pain	Diffuse abdominal pain, bloody diarrhea, nausea, vomiting, diffuse abdominal distension	UDE	Acute hemorrhagic colitis	DISCHARGE
Deb et al. 2021^16^	M	31	DM*, hypothyroidism, obesity	Hematemesis	UDE	Severe ulcerative esophagitis and non-bleeding gastric ulcers	DEATH
Author and year	Sex	Age	Comorbidities	GI symptoms	Diagnostic examination	Cause of bleeding	Outcome
	M	85	SAH, CKD, prostate cancer, sick sinus syndrome	Upper gastrointestinal bleeding	UDE	Gastric ulcer without bleeding, friable, necrotic, and with the possibility of perforation.	DEATH
	M	58	SAH, DM*	Melena	UDE	Gastric ulcers with non-bleeding craters but with a high chance of perforation	DEATH
Awwad et al. 2021^17^	M	30	NO	UGIB	UDE	Severe hemorrhagic ulcerative duodenitis	NR
Kumar et al. 2021^18^	M	71	NR	Hematochezia	Digital subtraction angiography	Active leak in the right iliac fossa supplied by the marginal branches of the ileocecal artery.	DISCHARGE

NR: not reported; DM: diabetes mellitus; SAH: systemic arterial hypertension; M: male; F: feminine; CA: cancer; UGIB: upper gastrointestinal bleeding; CKD: chronic kidney disease; UDE: upper digestive endoscopy; LDE: lower digestive endoscopy; CT: computed tomography; SBCE: small bowel capsule endoscopy; GIST: gastrointestinal stromal tumor. *The type of Diabetes Mellitus was not reported in the article. **The article does not numerically describe the patient’s age.

**Table 2:** Laboratory data from case reports and series.

Author and year	HB (g/dL)	HT (%)	PLAQ (mcL)	WBC (mcL)	INR	APTT (s)	FIBRINOGEN (mg/dL)	D-DIMER (ug/mL)
Mohamed et al. 2021^6^	8.9	41.3	93x10³	22.2x10³	2.3	24.7	198	>20
Argan et al. 2021^7^	15.1	NR	14.4x10^4^	6.4x10³	NR	NR	NR	0.6
	12.1	NR	23.2x10^4^	4.2x10³	NR	NR	NR	0.41
	13.4	NR	28.3x10^4^	5.7x10³	NR	NR	NR	1.66
Mitrovic et al. 2022^5^	10.6	NR	VR	VR	NR	NR	NR	1944
Li et al. 2021^8^	6.1	NR	20x10³	NR	1.45	NR	NR	NR
	5.5	NR	35x10³	NR	2.39	NR	NR	NR
Yamakawa et al. 2022^9^	12.4	NR	45x10³	4.9x10³	NR	NR	129	26.2
Kawabata et al. 2021^10^	12.2	NR	22.5x10^4^	13.2x10³	NR	47.7	NR	7.2
Krejčová et al. 2022^11^	8.1	NR	NR	NR	NR	NR	NR	NR
Merdad et al. 2021^12^	4	NR	NR	21.69x10^4^	NR	NR	NR	NR
Carl et al. 2021^13^	NR	NR	NR	NR	NR	NR	NR	NR
Jabłońska et al. 2022^14^	7.2	22	12.6X10^4^	4.51x10^5^	1.08	12.2	NR	NR
Carvalho et al. 2020^15^	NR	NR	24.4x10^4^	NR	NR	NR	NR	NR
Deb et al. 2021^16^	NR	NR	NR	NR	NR	NR	NR	NR
Awwad et al. 2021^17^	NR	NR	NR	NR	NR	NR	NR	NR
Kumar et al. 2021^18^	6.12	19.4	90x10^9^	NR	1.36	35.5	373	14.72

Hb: hemoglobin; HT: hematocrit; PLAT: platelets; WBC: total leukocytes; INR: internetional normalized ratio, APTT: activated partial thromboplastin time, NR: not reported.

**Table 3:** Summary data from original articles.

Title	Year	Methods	Results	Conclusion
Upper gastrointestinal bleeding in COVID-19 inpatients: Incidence and management in a multicenter experience from Northern Italy19	2021	COVID-19 positive patients with signs of acute UGIB in 6 hospitals in northern Italy were retrospectively included in the study. Patients admitted to the ICU environment were excluded. The Glasgow-Blatchford score was calculated, type of thromboprophylaxis or anticoagulant therapy was recorded, in addition to the severity of pneumonia. Upper digestive endoscopy (UDE) was performed when necessary.	Among 4,871 patients positive for COVID-19, 23 cases with UGIB were recorded (prevalence of 0.47%). 15 of these 23 patients had two or more comorbidities (78% hypertension or chronic heart disease, 48% DM, and 9% cirrhosis). 18 patients were on anticoagulant therapy. UDE was performed on 18 patients. The most common findings were peptic ulcer (44%), erosive or hemorrhagic gastritis (22%). The management of 52% of patients was conservative, with stabilization of Hb levels.	Upper GI bleeding was present in 0.47% of the sample and peptic ulcers were the most common finding. Conservative management may be an option in patients at high risk of complications
Gastrointestinal symptoms of 95 cases with SARS-CoV-2 infection3	2020	Single-center retrospective study; 95 patients diagnosed with COVID-19 were included; 6 patients with GI symptoms underwent gastroscopy.	Of 95 patients, 50 were women and 45 were men, with a mean age of 45.3 ± 18.3 years; 35 patients had comorbidities, such as hypertension in 16, DM in 6, malignant tumor in 5, chronic lung disease in 5, cerebrovascular disease in 4, and chronic kidney disease in 1; 58 patients manifested GI symptoms, 11 on admission and the other 47 during hospitalization. Diarrhea, anorexia and nausea were the most observed manifestations; 2 patients developed UGIB.	Understanding the susceptibility of the GI system to SARS-CoV-2 promotes more specialized therapy. The GIT can be a potential transmission route and is a target organ for COVID-19.

Regarding comorbidities, in 62.5% of cases, patients who developed GI bleeding had some type of coexisting disease^5^ ^-^ ^11^ ^,^ ^15^ ^,^ ^16^ ^,^ ^19^ ^,^ ^20^. In 68.7% of cases, patients developed signs of GI bleeding during hospitalization for COVID-19^5^ ^-^ ^11^ ^,^ ^13^ ^,^ ^16^ ^-^ ^18^; only 18.8% of patients arrived at the emergency room with symptoms of GI bleeding as the initial manifestation of viral infection^12^ ^,^ ^14^ ^,^ ^15^.

The presentation of bleeding of GI origin can manifest itself in two ways. In the studies included in this review, 37.5% presented in the form of upper gastrointestinal bleeding (UGIB)^8^ ^,^ ^10^ ^,^ ^16^ ^,^ ^17^ ^,^ ^19^ and 68.8% in form of lower gastrointestinal bleeding (LGIB)^6^ ^-^ ^9^ ^,^ ^11^ ^,^ ^13^ ^-^ ^18^. Some predisposing factors, such as antiplatelet therapy and/or anticoagulation, have been reported in 50% of cases^5^ ^,^ ^8^ ^,^ ^10^ ^,^ ^11^ ^,^ ^13^ ^,^ ^17^ ^-^ ^19^.

## DISCUSSION

### Pathophysiological mechanisms

The extrapulmonary manifestations of COVID-19 are well defined in the literature, especially the involvement of the GI system. The most discussed and scientifically supported pathophysiological mechanism is the use of ACE-2 receptors by the virus to penetrate GIT cells, carrying out viral replication. These receptors are expressed in abundance, not only in the respiratory system, but also in the GI, in the glandular cells of the gastric epithelium, small intestine, colon, and rectum. The replication of SARS-CoV-2 within GI cells will generate a cytopathic effect, with the release of inflammatory interleukins such as TNF-alpha, IL-1 and IL-6^5^ ^,^ ^9^ ^,^ ^11^ ^,^ ^13^ ^,^ ^15^ ^,^ ^17^ ^,^ ^20^. The role of IL-6 is highlighted in the hyperinflammatory process of COVID-19, as it is considered the main pro-inflammatory cytokine involved in this infection, and may be increased approximately 2.9 times in patients with complications resulting from COVID-19^9^ ^,^ ^21^. Chronic inflammation predisposes to the occurrence of pathologies that can lead to GI bleeding, such as esophagitis, colitis, rectocolitis, duodenitis, and ulcers^6^ ^,^ ^8^ ^-^ ^13^ ^,^ ^15^ ^-^ ^17^ ^,^ ^19^.

In addition to the important role of ACE-2 receptors, patients with COVID-19 may develop coagulopathy and vasculopathy, as well as resulting endothelial dysfunction and thrombosis^8^ ^,^ ^10^ ^,^ ^11^ ^,^ ^17^ ^,^ ^18^ ^,^ ^24^. COVID-19-associated coagulopathy (CAC) is characterized by increased D-dimer and fibrinogen levels, which can lead to thrombocytopenia. Furthermore, as a result of the viral cytopathic effect, macrophage activation syndrome (MAS) may develop, resulting in a thrombotic coagulation disorder. As the COVID-19 virus infects vascular endothelial cells, there is impairment in the antithrombotic mechanisms of the luminal surface due to the cellular damage and apoptosis^22^. As a result, hypoxic-ischemic injuries arising from thromboembolic complications can occur in the GIT, especially in the intestinal loops (which have abundant microvasculature), resulting in GI ulcerations and bleeding^8^ ^,^ ^10^ ^,^ ^13^. Furthermore, due to vascular fragility resulting from CAC, there may be bleeding in more fragile vascular branches, such as the rectal and the marginal branches of the ileocecal artery^6^ ^,^ ^18^.

The case of Carll et al.^13^ brings a relationship between COVID-19 infection and the reactivation of latent infections, in this case by cytomegalovirus (CMV), as a result of severe lymphocytopenia triggered by SARS-CoV-2, manifesting acute hemorrhagic enterocolitis. This depletion of lymphocytes is potentially associated with the dysfunction of the reticuloendothelial and hematological systems caused by the virus, as well as the sequestration of lymphocytes by the inflammatory cytokines produced in an exacerbated manner. In this case, GI bleeding is described as resulting from this viral cytopathic effect of COVID-19, aggravated by CMV co-infection, generating persistent inflammation in the mucosa.

### Anticoagulation and antiaggregation therapies

In some management protocols for patients with COVID-19, there is an indication to carry out anticoagulation and thromboprophylaxis therapies, to avoid greater damage due to CAC, as well as to enable support with extracorporeal membrane circulation (ECMO)^8^ ^,^ ^9^ ^,^ ^13^ ^,^ ^17^. By carrying out these therapies to avoid thromboembolic events, there is a predisposition to more profuse bleeding, which means that in lesions in an inflammatory/hemorrhagic process, bleeding is greater, leading to hemodynamic instability in the patient more quickly^8^ ^,^ ^10^ ^,^ ^11^ ^,^ ^13^ ^,^ ^17^ ^-^ ^19^. Monitoring D-dimer and fibrinogen in patients who develop a severe form of the disease can help in the early diagnosis of events resulting from the hypercoagulable state, since these two laboratory data are associated with a high risk of developing micro and macrocirculatory thrombosis, justifying anticoagulation and antiaggregation therapies at the most opportune moment, avoiding profuse GI bleeding^10^ ^,^ ^11^ ^,^ ^23^ ^,^ ^24^.

### Types of bleeding and injuries to the GI mucosa

The manifestations of gastrointestinal bleeding are divided into UGIB and LGIB, which become evident by the presence of signs such as melena, hematemesis, hematochezia, and enterorrhagia. Other symptoms that may be associated with the presence of GI bleeding are diarrhea, pain, and abdominal distension^2^ ^-^ ^4^ ^,^ ^19^.

The deleterious impact of SARS-CoV-2 infection on GIT cells can manifest in different ways, and GI bleeding is related to higher mortality during hospitalization^25^ ^,^ ^26^. Identification of the type of injury allows targeted treatment, avoiding complications and improving patients’ prognosis. The most frequently described lesions were gastric ulcers^10^ ^,^ ^16^ ^,^ ^19^ and duodenal/jejunal ulcers^8^ ^,^ ^12^, but there have been reports of ulcers in the colon and rectum^9^, erosive/hemorrhagic gastritis^19^, ischemic ulcerative colitis^11^, hemorrhagic enterocolitis^13^, acute hemorrhagic colitis^15^, esophagitis^16^, hemorrhagic ulcerative duodenitis^17^, injury to marginal branches of the ileocecal artery^18^, and injury to rectal branches^6^.

The occurrence of GI bleeding in some patients with COVID-19 followed by hemodynamic instability is deemed serious, as this circulatory imbalance is considered a complicating factor in the infectious condition^6^ ^,^ ^8^ ^,^ ^11^ ^,^ ^24^.

### Patient management

There are several guidelines for the diagnostic and therapeutic management of GI bleeding. The Japanese guidelines state that patients with UGIB should undergo UDE within 24 hours of the onset of symptoms. The European guidelines, on their trun, state that performing endoscopic procedures in patients with UGIB and COVID-19 without hemodynamic instability is at medical discretion^10^.

Computed tomography (CT) has been described as one of the diagnostic methods^5^ ^,^ ^7^ ^,^ ^12^, but the most used and recommended is endoscopy, especially esophagogastroduodenoscopy, responsible for the diagnosis in 43.8% of cases^5^ ^,^ ^9^ ^,^ ^10^ ^,^ ^15^ ^-^ ^17^ ^,^ ^19^. Angiography_6,18_, laparotomy^11^ ^,^ ^14^, colonoscopy^9^ ^,^ ^11^ ^,^ ^13^, and small bowel capsule endoscopy (SBCE)^8^ were also described as diagnostic tests, but are less used.

At the beginning of bleeding, a bolus of proton pump inhibitor (PPI) has been described as an effective measure to reduce bleeding, being one of the recommended conservatives treatments^5^ ^,^ ^10^ ^,^ ^12^ ^,^ ^19^. Another medication, used by Awwad et al.^17^ as an alternative to stop bleeding, was oral budesonide, a glucocorticoid, acting to reduce local inflammation, migration of blood cells, and protein exudation. In cases of lack of satisfactory response to more conservative techniques, endoscopic treatment should be considered^12^ ^,^ ^23^. Embolization of arteries by interventional radiology and the use of endoscopic hemospray to achieve hemostasis are techniques described that have been successful^6^ ^,^ ^16^ ^,^ ^18^.

The last line of treatment is the surgical approach, considered in those patients with bleeding refractory to PPIs and endoscopy, or in those who present hemodynamic instability that is not controlled with fluid replacement^8^ ^,^ ^12^ ^,^ ^23^.

It is worth noting that extrapulmonary manifestations of COVID-19 can occur concomitantly with pulmonary involvement or in isolation, with aggression in more than one system being more related to severe forms of the infection^2^ ^,^ ^3^ ^,^ ^24^. In the data collected in this review, 43.75% required admission to the ICU^6^ ^-^ ^9^ ^,^ ^12^ ^,^ ^14^ ^,^ ^17^, which may indicate that gastrointestinal involvement with bleeding leads the patient to deteriorate, requiring intensive monitoring. 

### Study limitations

This review has some limitations, since due to the methodology it is not possible to define the best diagnostic method and the most effective treatments based on clinical manifestations. Prospective studies are needed to more specifically identify the impacts of COVID-19 on the gastrointestinal system for the occurrence of bleeding and more targeted management of affected patients. Filling these knowledge gaps will allow for better quality care for patients presenting with gastrointestinal bleeding related to SARS-CoV-2 infection. 

## CONCLUSION

The presence of gastrointestinal bleeding in patients with COVID-19 is established in the literature, since the pathophysiological mechanisms of COVID-19 directly affect the GIT. Identifying signs and symptoms early with the help of laboratory data and recognizing the most common injuries allows for the management of these patients, which is essential for reducing morbidity and mortality.
